# Employee Perceptions About Participation in Decision-Making in the COVID Era and Its Impact on the Psychological Outcomes: A Case Study of a Cooperative in MONDRAGON (Basque Country, Spain)

**DOI:** 10.3389/fpsyg.2022.744918

**Published:** 2022-02-04

**Authors:** Aitziber Arregi, Monica Gago, Maite Legarra

**Affiliations:** Faculty of Business Studies, Mondragon University, Gipuzkoa, Spain

**Keywords:** employee participation, COVID, psychological wellbeing, MONDRAGON, degeneration, regeneration

## Abstract

This research aims to study possible effects or impacts of COVID-19 in the context of a democratic organizational system analyzing how COVID-19 has influenced employees’ perception of their participation in decision-making and its impact on some psychological outcomes and emotions. COVID-19 has accelerated the process of implementation of new frameworks at work (digitalization, teleworking, new skills, and abilities) that have generated the modification of culture and employee management practices. Our hypothesis are, on the one hand, that COVID-19 has generated changes in participation structures and internal communication mechanisms, having to make modifications not to deteriorate the perception of employees about their participation in decision making. On the other hand, COVID-19 has generated changes in the psychological outcomes and emotions of the employees. In the study, we analyze a cooperative belonging to the MONDRAGON cooperative group, where participation in decision-making and ownership is in its DNA. Through qualitative (5 focus groups) and quantitative (short questionnaire) methodologies, involving 42 employees, we investigate firstly, how COVID-19 has affected perceptions about participation in decision-making analyzing what role has played internal communication in these perceptions. Secondly, we investigate how COVID-19 has affected psychological outcomes and emotions. In this case, the perceptions arising from participation in decision-making focus on the assessment that participators make of the governance channels and the day-to-day meetings. Therefore, their appropriateness seems to be a key factor in the perception of participation in the COVID-19 era. Differences have been detected between the perceptions of blue and white collar employees. Such differences have also been founded in the psychological outcomes and emotions. Although this is a single case study, the analysis carried out provides elements of reflection to modify and restructure the decision-making and participation mechanisms, adapting them to the needs of blue and white collar employees in order to “guarantee” the expected outcomes.

## Introduction

COVID-19 has provoked a new economic crisis that has forced many organizations to close, but it has also accelerated the implantation of new approaches to work and new human resource management practices, such as digitalization and remote working, thus reinforcing the need to acquire new knowledge and skills in one’s job in order to meet the new strategic goals of the business (Carnevale and Hatak, 2020; DeFilippis et al., 2020; Xifra, 2020; Yawson, 2020). In addition, there is a new discourse regarding the need to modify the work culture of organizations (Spicer, 2020) which moreover may lead to reassess employees’ participation in organizations (Child, 2021). In this new paradigm, the mechanisms of participation and the structures of decision-making seem to be rethought and adapted to the new reality. Structures of governance and communication and information policies that facilitate participation may be questioned and redefined, and this may affect the way the principle of democratic organization is exercised within cooperatives. Moreover, COVID-19 has an effect to the emotions and psychological outcomes, not only as citizens, but also as employees (e.g., Tang et al., 2020).

In this regard, a case study has been carried out of the cooperative ULMA Architectural Solutions (UAS) belonging to MONDRAGON, in which the influence of COVID-19 on employee’s perception of their participation in decision-making (PDM) and internal communication (IC) has been analyzed. In addition, employees were asked about their perceptions regarding psychological outcomes affected, and the emotions experienced during the COVID-19 pandemic. Some years before, Arregi et al. (2018) carried out a quantitative study in the same organization, UAS, in order to examine perceptions of organizational atmosphere and joint ownership, and they reinforced the fundamental roles of information-sharing and participation in enterprises with shared ownership.

This article allows us to examine, from different angles, the impact of COVID-19 on an organization where participation is a central element due to its nature as a cooperative. As far as we know, this is one of the few studies, or even the first one, in which democratic processes and IC practices in a MONDRAGON cooperative have been examined at the level of employee-owners in the COVID-19 era.

Additionally, it permits an analysis of the point of view of both white and blue collar employees, a perspective that is unusual in the scientific literature, but of great interest given that the two groups differ considerably in terms of their perceptions of the workplace (Hu et al., 2010). The study carried out by Arregi et al. (2018) found key distinctions between blue collar (BC) and white collar (WC) workers’ experiences and perceptions in UAS.

Our first research aim will be the analysis of the employees’ perceptions about PDM. At organizational level, employee participation has a special relevance as a lever of business innovation. In today’s global economy, organizations are realizing that people are at their neurological core (Noe et al., 2017). The approach related to the participative organizational model stands on the idea of placing the person at the center of the organization. In this regard, a new form of internal governance and employee participation may prove to be decisive. Wegge et al. (2010), based on Weber et al. (2009), suggest that Organizational Democracy (OD) is the most radical form of employee participation. This concept includes direct or representative joint consultation, co-determination and self-determination in organizations (Heller et al., 1998) and employees often hold a share in their organization‘s equity capital. We add to the OD literature by gathering contributions from Strategic Human Resources Management (HRM) theory, which endorses High Performance Working Systems (HPWS) which in turn, encompass employee PDM, among other aspects.

Within participative models, cooperatives are by nature the organizations with the greatest potential to integrate involved, active and participative people into their projects, as participation is a basic principle of their business formula (Altuna Gabilondo, 2008). Cooperatives differ substantially from the conventional companies in their basis purpose, property rights and decision making processes and “they are in tune with a more participatory and democratic society” (Forcadell, 2005, p. 255). Indeed, in the cooperatives within MONDRAGON, participation (according to the principles of democratic organization and PDM) is the spine of the group’s cooperative experience (Altuna Gabilondo, 2008). The philosophy of cooperativism, particularly as manifest in worker cooperatives, gathers economic and democratic rights and the ownership has economic and socio-political senses (Cheney, 2006). MONDRAGON is one of the most studied success stories in the scientific literature (Whyte, 1995; Forcadell, 2005; Basterretxea and Albizu, 2011; Freundlich et al., 2013; Heras-Saizarbitoria, 2014; Gallego-Bono and Chavez-Avila, 2016; Azkarraga and Cheney, 2019). However, PDM in the cooperatives in general, and also in MONDRAGON, has presented some “dilemmas and paradoxes” focused on the degeneration and regeneration of the cooperatives that scientific literature gathers (e.g., Greenwood and González, 1991; Stohl and Cheney, 2001; Cheney, 2006; Azkarraga et al., 2012; Agirre et al., 2014; Errasti et al., 2016, 2017; Bretos et al., 2017, 2020).

MONDRAGON is a well-known example of an organization in which employees of the cooperatives are also “joint owners” (Forcadell, 2005) of the organization, that is, organizational democracy is conceived as “each member has one vote regardless of their capital contribution.” In cooperatives, there exist governance channels through which democratic principles are put into practice, where communication takes place and participation is made possible; namely, the General Assembly, the Governing Council, the Audit Committee, the Social Council and the Management Council.

In MONDRAGON, IC and PDM cannot be separated from each other in this case study. According to the social and business policy of MONDRAGON for the period 2009–2012, IC was related with participation, involvement, cohesion and motivation of employees, thus conceiving IC as much more than simple channels and tools (Belategi et al., 2019). Moreover, the MONDRAGON Corporate Management Model in 2013 emphasized three aspects of promoting democratic governance and participation: self-management, communication and corporate development (Bretos et al., 2020). The research is based on the idea that IC is an essential foundation for PDM to function. While IC can be practiced without democratic decision-making (such as, for example, in hierarchical companies), mechanisms of IC need to be functioning in order for PDM be exercised.

As a result of the pandemic some organizations had to find new ways of communicating and organizing due to the social distancing, digitalization or remote working. The challenges in this field seem to be changing and this topic is relevant as the health crisis forces organizations to adapt work processes and it has an enormous impact on the individual as an employee (Li et al., 2021). In this situation, democratic governance channels and methods together with IC and information policies may be reassessed.

In this new paradigm where the COVID-19 has affected psychological outcomes, as numerous recent studies have addressed (i.e., Adhitama and Riyanto, 2020; Carnevale and Hatak, 2020; Bulińska-Stangrecka and Bagieńska, 2021), our second research aim is to analyze the impact of the COVID-19 in psychological outcomes and emotions, although we recognize that the empirical study does not allow to measure the impact of PDM on psychological outcomes arising from COVID-19. However, this research allows to theorize about the relations among all the factors analyzed in the study.

Within all this context, the present research aims to illuminate how COVID-19 has influenced: (1) employees’ perceptions of their PDM through formal governance channels and IC mechanisms; and (2) psychological outcomes and the emotions experienced by employees; with special attention given to whether there is a divergence between BC and WC employees.

### The Case: ULMA Architectural Solutions

ULMA Architectural Solutions is a small-to-medium-sized employees’ cooperative based in the Basque Country, Spain, with a population of approximately 2.19 million in 2020. It’s a region with a strong industrial past, and, although COVID-19 has had a negative impact on industry, unemployment remained at 11.2% and the working population increased by 1.5% in the fourth quarter of 2020 (Eustat, 2021). Comparing it with other regions of Spain, it has not suffered the effects of the health crisis to the same extent.

The firm was founded in 1990 as a worker cooperative. It designs and manufactures prefabricated products for the construction of drainage and external wall systems, architectural precast and ventilated facades. It’s workforce has averaged approximately 254 people in 2021, of which approximately 82% are employee-members, with 115 employees BC and 139 WC.

ULMA Architectural Solutions belongs to MONDRAGON, which is an integrated network of over 95 cooperatives and approximately 135 affiliated organizations. Together, these firms generated €12,320 million in sales and had a workforce of over 81,000 people in 2019.

In cooperatives, participation can occur in three differentiated areas; PDM, economic participation, and participation in ownership, as will be explained below.

In MONDRAGON cooperatives, the members’ participation is fostered in three areas: in the ownership of the organization, as they contribute to the cooperative economically and therefore, they become owner-members; in the so-called “economic participation,” as they receive their corresponding economic reward; and in the decision-making of the cooperative through their voice and/or vote by means of the institutional (formal) democratic structures (governance channels) provided by the cooperatives. These governance channels provide the members with the mechanism for individual participation (either directly or through their representatives) and allow and promote PDM.

1. *The General Assembly*: according to article 1 of the law of cooperatives of the Basque Country, “this is a meeting of the employee-members, held to deliberate on and make decisions about matters of their competence.” It is the supreme body of the cooperative (Larrańaga, 2005) and one that expresses the communal will of all the participating employee-members (Altuna Gabilondo, 2008). Its functions are: to adopt strategic plans of action and annual management plans, to name and revoke the members of the Governing Council, and to adopt agreements regarding the cooperative’s legal system (i.e., statutes or internal rules of procedure). The General Assembly is composed by all the members of the cooperative where each of them has one vote, regardless of the contribution to the capital done.
2. *Governing Council*: this is the representative, managing and governing body of the cooperative (Larrańaga, 2005; Altuna Gabilondo, 2008; Altuna and Grellier, 2008), and its role is to exercise all the faculties that by law or according to the statutes are not expressly the function of the other corporate bodies. Its members are appointed by all the members of the cooperative in the General Assembly. Its functions are divided into (a) social functions, (b) legislative and institutional functions, and (c) functions related to financial management.^1^
3. *Audit Committee*: it is the supervising body, whose functions are surveillance, mediation and transmission of information. Other functions are the control of accounts and audits: review of the annual accounts, end-of-year balance sheets, etc. (Larrańaga, 2005). It oversees the process of election and designation by the General Assembly of the members of the other bodies. Its members are elected by all the owner-members in the General Assembly.
4. *Social Council*: this is an advisory representative body of the employee-members without any decision-making capacity, as it merely fulfils an advisory, informative and consultant role in all aspects affecting labor relations. It is the main channel of dialogue for the employees. It is common practice in cooperatives for its members to be named by the different departments or sections; in this way, the representatives of the Social Council represent the employees of the department or section to which they belong. The meetings that each representative holds with his/her department/section are known as social council constituents’ meetings (*consejillos*) (Larrańaga, 2005).
5. *Management Council*. This is the executive body and is formed by managers and directors of the cooperative. It coordinates the functions of the management team and the advisory role of the Governing Council. Its main function is to coordinate and stimulate the day-to-day business management; i.e., to advise Management in aspects of business promotion and development, making forecasts and carrying out planification, and proposing actions to be taken with respect to business activity. It is responsible for monitoring the work of the executive branch of the company (Larrańaga, 2005) and its members are appointed by the member’s representatives on the Governing Council.

Apart from the above-mentioned governance channels, diverse types of meetings serve as channels of communication in UAS, as they do in other types of businesses. Meetings are one of the most known forms of IC (Ongallo, 2001; Enrique, 2007), and in cooperatives they include meetings of collaborators, departmental/area meetings, etc.

Since the aim of the article is to analyze the impact of COVID-19 in UAS, we should highlight that, due to the declaration by the Spanish government of a national state of alarm on 14 March 2020, UAS ceased its activity for 2 weeks, and thereafter modified its work practices. WC employees began working from home and BC employees returned physically to their workplace and began to work on a flexible timetable.^2^ In the case of those on temporary contracts, 20 employees were let go with a commitment to contract them again as soon as there was work, which occurred in less than 2 months.

## Theoretical Framework

Employee participation has been widely studied, among others, within the fields of OD and HRM. In these fields, the academic approach to the concept of participation has different perspectives.

According to authors such as Harrison and Freeman (2004), Kerr and Caimano (2004), Weber et al. (2009), and Yazdani (2010) OD is an approach related to the management where employees are involved in the decision-making and management process. OD describes an organizational climate where employee participation is institutionalized, broad-based and ongoing (Wegge et al., 2010). Therefore, OD comprises the idea that employees participate in the organizational and governance processes (Harrison and Freeman, 2004) and where the majority of employees “participate in the form of institutionalized and binding involvement (mandatory joint consultation) or decision-making (i.e., codetermination with equal representation or collective self-determination), refer to tactical or strategic decisions; considers issues at the organizational level (a large unit, plant, corporation, etc.); participate either directly (within meetings or general assemblies) or indirectly through their representatives who are elected or appointed into a representative board/committee; and often hold a share in their organization’s equity capital” (Wegge et al., 2010, p. 162; Weber et al., 2020).

Focusing on organizational participation and its definition, Wilpert (1989, p. 79) states that “participation is the totality of forms, that is, direct (immediate, personal) or indirect (via representatives or institutional means) and intensities, ranging from negligible to comprehensive, through which individuals, groups, collectives ensure their interests through self-determined choices of possible actions.”

At this respect, some authors differentiate three areas in which participation can occur: PDM, economic participation and participation in ownership (Rodríguez-Sañudo Gutierrez, 1979; Hermel, 1990; Albizu et al., 2005).

1. PDM includes employee participation in day-to-day management activities; sense-making; and governance decision-making, which refers to decisions related to overall control through formal governance bodies (Baniandrés and Larrinaga, 2013). This classification is in line with the distinction found in Eurofound (2013), where PDM is divided into three levels: one’s job; the department or section the employee belongs to; and strategic decisions. Bernstein (1976b, p. 492) distinguished between: “the degree of control employees enjoy over any particular decision; the issues over which that control is exercised; and the organizational level at which their control is exercised” and settled that PDM was the first necessary component of democratic workplaces (Bernstein, 1976a).
2. Economic participation refers to the economic rewards employees receive: salary, recognition, profit- or gainsharing, or any other economic benefit.
3. Participation in ownership refers to the relationship of the employee with respect to ownership of the enterprise, as a shareholder or as employee-member.

As said before, participation has also been studied from the HRM field. The focus has been on the importance of the person according to the resource-based theory that stressed the importance of internal resources of an organization (Barney, 1991) in generating a competitive advantage (Wright et al., 1994; Jackson and Schuler, 1995; Chadwick and Dabu, 2009). In this sense, employees can be considered fundamental in generating this advantage (Wright and McMahan’s, 1992), especially if they are aligned with the organization’s strategy (Begin, 1991; Capelli and Singh, 1992; Jackson and Schuler, 1995).

The Strategic HRM theory holds that what are known as HPWS increase the knowledge, abilities and skills of people, thus empowering them, enhancing their motivation (Becker and Huselid, 1998; Delery and Shaw, 2001), and creating the competitive advantage that has proved to be so necessary in organizations in recent times (Xian et al., 2019; Miao et al., 2020; Wattoo et al., 2020).

There is no consensus among academics regarding the definition of HPWS, or about what practices they should involve (Boxall and Macky, 2014). However, one of the practices usually included within the system is PDM (Huselid, 1995; MacDuffie, 1995; Appelbaum et al., 2000; Combs et al., 2006; Guthrie et al., 2009; Boselie, 2010; Zhang et al., 2013; García-Chas et al., 2014; Ramdani et al., 2014). Apart from being the first necessary component for a workplace democratization (Bernstein, 1976a), PDM can be considered one of the stalwart practices of HPWS, and one which allows businesses to achieve competitiveness (Messersmith et al., 2011).

In this context, cooperatives are by nature the organizations with the greatest potential to integrate involved, active and participative people into their projects. In fact, the three areas of participation mentioned above, are present in the cooperatives. Moreover, participation is a basic principle of their business formula and in the cooperatives within MONDRAGON, participation is the spine of the group’s cooperative experience (Altuna Gabilondo, 2008) and one of its identifying features (Agirre et al., 2015). However, as mentioned before, the participation in cooperatives has some lights and shadows that literature gathers. Bretos et al. (2020) offer a detailed revision of the debate on degeneration and regeneration, where “the organizational degeneration implies that employee involvement in decision making is diminished in favor of control by a managerial elite or technocracy,” and the regeneration thesis implies that “democracy is reinvented.” In particular, Bretos et al. (2020) revealed that the life cycle of international cooperatives evolves differently to those small and medium-sized cooperatives that operate exclusively locally and that cooperatives are able to activate processes of organizational change tended to revitalize cooperative values and practices. Notwithstanding the above-mentioned, they found that degenerative and regenerative tendencies can occur simultaneously. In light of the conclusions and questions arisen from such study and in this scenario of COVID-19, we consider it interesting to analyze whether the pandemic had lead a cooperative such as UAS to develop any symptom related to a degeneration or regeneration process.

As mentioned in the introduction, in the case of MONDRAGON, IC is related with participation. In accordance with Belategi et al. (2019), the principle of PDM, implies a progressive development of self-management and, consequently, the participation requires the development of adequate mechanisms and channels of participation and transparency of information.

Wilkinson et al. (2010) define employee participation as the range of mechanisms used to involve the employee in decisions at all levels of the organization where information and communication are considered to be main components of the process. They proposed the “escalator of participation” where it can be observed a progression upwards from information to make decisions (information, communication, consultation, codetermination, and control). So, in this sense, IC is a key aspect to develop employee participation.

Internal communication is a practice by which information is collected, shared and distributed to ensure that employees understand the organization’s objectives and goals (Verčič et al., 2012; Uysal, 2016; Smaliukienė and Survilas, 2018). According to García Jiménez (1998) and Del Pozo (2000), investment in IC generates participation, identity, unity and a sense of belonging within the organization. Kovaitė et al. (2020) affirm that IC plays a key role in maintaining employees informed about the plans, vision and business ideas of the organization and it encourages them to participate in the decision-making process. In this way, IC can be considered an indispensable tool for organizations (Carrascosa, 1992; Peters, 1995; Sánchez and Shimón, 1997; Elías and Mascaray, 1998; Gonzalo, 2002; Jaén Díaz et al., 2006; Saló, 2007; Welch and Jackson, 2007; Hargie and Tourish, 2009; Tessi, 2012; Vilanova, 2013) and to develop employee participation (Belategi et al., 2019).

Moreover, Belategi et al. (2019) endorse the importance of listening in IC in one cooperative in MONDRAGON, based on Tessi (2012, p. 65), who says that “one of the most important and strategic decisions to be made in terms of IC is to actually prioritize listening above everything else.” In line with this, in the 89th Session of the Communication Forum, held in Spain in 2020, under the title “IC in times of COVID: a turbulent present and a gaze toward tomorrow” (translation of the original title), a key aspect of human resources departments seems to be a drive to ensure IC based on the ability to listen, leadership and empathy.

The COVID-19 pandemic has modified the way organizations act and work (DeFilippis et al., 2020) and has also accelerated the implantation of new approaches to work, such as digitalization and remote working (Carnevale and Hatak, 2020; Xifra, 2020; Yawson, 2020). Indeed, there is a new discourse regarding the need to modify the work culture of organizations (Spicer, 2020). The pandemic has affected the means and techniques of IC (Sanders et al., 2020; Xifra, 2020) and has highlighted the need for new styles of management of IC, with traditional forms of IC becoming digital (Ruck and Welch, 2012; Ewing et al., 2019), in the form of social networks, streaming etc. According to Gallup (2020), IC has allowed people to work in a more productive manner and to maintain their work commitments in a context of uncertainty, ambiguity and changes.

In scientific terms, it is interesting to study the impact of COVID-19 on democratic structures and practices in a cooperative belonging to MONDRAGON. This research will be done through the analysis of the impact of COVID-19 on the employees’ perceptions about PDM, governance channels and IC. Changes in these indicators may prevent or foster tendencies of the generation or degeneration in cooperatives.

We are interested in performing this analysis from the perspective of WC and BC employees, an approach that is unusual in the scientific literature, but of great interest given that the two groups differ considerably in their perceptions. Many of the cooperatives within MONDRAGON are manufacturing businesses where employees perform jobs of a diverse nature and have qualitatively different experiences and perceptions concerning their work.

It is relevant to underline that the literature has not taken into consideration the importance of the type of job/professional group to which employees belong when explaining employees’ perceptions of their job and organization (Blauner, 1964; Dubin et al., 1975).

Historically, the term known colloquially as WC has been used to refer to office employees, while that of BC has been employed for manual employees (Shirai, 1983). Hopp et al. (2009) noted that BC employees carry out physical and routinary tasks while WC employees perform any task that requires intellect or creativity. However, as Ramirez and Nembhard (2004) pointed out, this distinction does not necessarily mean that there are jobs which are purely BC or WC.

It is relevant to consider this classification of employees, as the literature shows that, for diverse reasons, including frequently lower levels of job complexity and autonomy, higher levels of physical effort and other less favorable working conditions, worse career prospects, less formal education, factory/manual and other BC employees’ perspectives are generally found to be more negative than those of WC employees; in fact, research suggests that their very conceptions of work, satisfaction and career and related experiences are notably different from those of the other group (Hu et al., 2010).

UAS is an industrial cooperative whose percentages of BC and WC employees are 45 and 55%, respectively. Arregi et al. (2018) in the same cooperative observed that, when questioned, employees’ perceptions of the organizational atmosphere and of cooperative ownership beliefs and behavior were strongly associated with whether they were BC or WC. Consequently, this classification has been taken into account as far as possible in the present research.

In summary, working conditions for employees have been altered drastically in COVID-19 era; from working on the premises of the organization to working on-line or under new company policy and procedures that reduce physical contact among employees (Carnevale and Hatak, 2020). This represents a great challenge to employee participation (spine of the experience of MONDRAGON), with a subsequent need to modify the channels and mechanisms of decision-making and communication. This context leads us to the first research question:

*To analyze how COVID-19 has affected employees’ perceptions of their PDM and how governance and communication channels have been adapted to satisfy the new needs arising out of the current situation, differentiating between BC and WC.*

Beyond the effect of COVID-19 on PDM, it has clearly influenced employees’ psychological situation. Some of the most studied psychological outcomes in the literature are satisfaction, commitment, trust and motivation (Meyer and Allen, 1991; Meyer, 2001; Colquitt et al., 2007; Allan et al., 2019). A year after the first global lockdown, diverse studies have analyzed the psychological consequences the pandemic has had for employees in the workplace. In this new paradigm, there is a clear effort on the part of scientific research to assess the psychological impact of COVID-19 on the well-being of employees (Carnevale and Hatak, 2020; Lee et al., 2021; Meyer et al., 2021; Möhring et al., 2021); on their motivation and commitment (Adhitama and Riyanto, 2020; Chanana, 2020; Kaushik and Guleria, 2020; Mani and Mishra, 2020; Risley, 2020; Yuan et al., 2020; Jung et al., 2021); on their satisfaction (Bulińska-Stangrecka and Bagieńska, 2021; Ojo et al., 2021); trust (Gillespie et al., 2020); health/stress (Hamouche, 2020; Teng et al., 2020; Reizer et al., 2021), or on their sense of uncertainty (Tang et al., 2020); several of which aspects were analyzed in the previous crisis of 2008 (Tonkiss, 2009; Biswas and Biswas, 2010; Markovits et al., 2014; Závadský et al., 2015; Meyer et al., 2018; Charles et al., 2019).

Relationship between PDM and psychological outcomes has been largely studied. We find interesting to overview the literature corresponding to such relationship. This review offers arguments/evidences to be taken into account, not only by organizations to foster employee participation, but also by employees to participate.

Several studies in the OD field have showed that employee participation is related to psychological outcomes such as satisfaction, commitment, motivation, organizational civism and other similar (see some references in Weber et al., 2020). Jarley et al. (1997) stated that democratic governance is considered key to greater organizational effectiveness and is mandatory to achieve higher levels of innovation and performance (Manville and Ober, 2003).

Within the HRM field, many studies have explored the relation between HPWS (where PDM is one of the practices taken into account more commonly) and competitiveness (Paauwe, 2009; Guest, 2011; Jiang et al., 2012; Paauwe et al., 2013; Elorza et al., 2016). Other studies have analyzed the relationship between HPWS and individual employee psychological outcomes, such as satisfaction, commitment and well-being (García-Chas et al., 2015; Huang et al., 2016, 2018; Teo et al., 2020).

If we focus on organizations where the ownership is broadly shared, literature suggests that such organizations tend to generate more positive psychological outcomes than those enterprises where the ownership is not broadly shared (Long, 1978, 1980; Wilson and Peel, 1991; Van Dyne and Pierce, 1993; Kruse, 2002; Höckertin and Härenstam, 2006; Reeves, 2007; McQuaid et al., 2012; Poutsma et al., 2015), although there are studies that reach the opposite conclusion (Arando et al., 2015).

With regard to cooperatives, Ortega (2019) concludes that academic literature tends to confirm that cooperatives have greater resistance to maintaining activity and employment in periods of economic recession (Pomares Hernández and Grávalos Gastaminza, 2001; Díaz Foncea and Marcuello Servós, 2010; Cantarero Sanz et al., 2013; Morandeira, 2014; Sala Ríos et al., 2014, 2015; Villafańez, 2014; Bretos and Morandeira, 2016; Perad, 2016; Soto Gorrotxategi and Diaz Molina, 2018).

Numerous studies have demonstrated the positive aspects and economic or social benefits of financial participation, for employees (Rhodes and Steers, 1981; French and Rosenstein, 1984; Oliver, 1984, 1990; Klein, 1987; Dong et al., 2002; Gamble et al., 2002; Kruse, 2002; Kuvaas, 2003; Höckertin and Härenstam, 2006; Kruse et al., 2008, 2010; Employee Ownership Association [EOA], 2010), for the company (Employee Ownership Association [EOA], 2010), and for the broader community (Kruse et al., 2010).

Although the above literature only focus on economic participation and ownership, some authors argue that is the combination of different areas of participation within companies that can help to achieve different psychological results. Klein (1987) proposed the Instrumental Satisfaction Model, by which capital ownership is widely shared and accompanied by other organizational policies and practices related in particular to information-sharing and employee PDM. This combination of policies and practices and shared ownership will lead employee-members to be satisfied with their ownership and will bring other positive outcomes to the company.

Therefore, employee participation is one of the tools with most potential available to companies, and through more participative organizational models, employees can participate in the company, from decision-making to ownership (Elorza et al., 2016), allowing the best organizational and psychological results to be achieved.

In addition, several studies highlight a positive relation between an adequate policy of IC and various psychological outcomes, such as employee satisfaction (Bustamante, 2013), motivation (Opitz and Hinner, 2003; Bustamante, 2013), the identification of employees with the company (Smidts et al., 2001), and employee well-being (Welch, 2011; Ruck and Welch, 2012; Verčič et al., 2012; Ruck, 2020).

In this regard, although we will not analyze the relation between the changes of perceptions related to PDM and psychological outcomes derived from the COVID-19 era, we have considered it interesting to address the second research question:

*To analyze how COVID-19 has had an effect on psychological outcomes and the emotions experienced by employees during this period.*

## Materials and Methods

With the aims explained above, we have used a qualitative and quantitative design for this study.

The research was carried out at UAS, an employees’ cooperative within MONDRAGON, as already mentioned (see section “The Case: ULMA Architectural Solutions”).

To explore the gaps detected in the theoretical framework, we considered that a case study was the methodology that would best fulfill the needs and objectives of our research. As indicated by Yin (2013) and Maxwell (1996), the case study methodology is the most appropriate when the research questions require a profound exploration of a social phenomenon (such as COVID-19), when the focus of the research is to understand said phenomenon and its processes, and when it is important to analyze the how and why of the phenomenon.

One of the limitations of this methodology is that the interpretations of data are based on the impressions of the researchers and, therefore, cannot be extrapolated to other contexts. In order to reduce the impact of this limitation, we have used different techniques to triangulate the evidence and not base conclusions solely on the researchers’ interpretation. In addition, we have applied validity criteria such as the use of multiple sources of evidences, the maintaining of the chain of evidence that allows to trace the data and the revision of the draft of the results by a key informant in UAS (Yin, 1998). On the other hand, it has not been our intention to generalize the results or conclusions drawn in this article, as each business has its peculiarities and nuances that make it unique and impede the extrapolation of results to other companies. Nevertheless, the results obtained in this research through the case study model try to facilitate the construction of arguments, interpretations and insight into employee experiences, all of which can be of use to other organizations that are preoccupied about how COVID-19 may affect the participation of its employees and IC, and which are conscious of the concerns of this new paradigm. With this study we invite other academics for further studies in the field of the participation in the new era after COVID-19, either in cooperatives or other democratic organizations.

In the qualitative methodology, the focus group, described below, is the main method used to address the research questions. For the quantitative methodology, on the other hand, an online questionnaire was employed with the aim of encouraging the employees’ participation by enquiring about their opinions and perceptions. The two methodologies are considered to be complementary when seeking responses to the research questions put forward.

That said, the principle technique of data collection in the study has been the employment of a focus group. The technique consists of organizing meetings of small or medium-sized groups in which participants discuss one or various subjects in an informal environment, with guidance from researchers (Hernández et al., 2010). In this regard, the data collection has followed a case study protocol designed by the researcher in order to deal with the reliability of the study (Yin, 1998).

Five focus groups were carried out on the 12th (1 focus group), 13th, and 14th (2 focus groups each day) October 2020. The number of participants in each focus group varied, from 6 to 11 people, with a total of 42 participants among the five groups. By request of the company, the groups lasted an average of 30 min. The duration of the focus and the number of participants can be seen as a limitation of the study. However, the researcher tried to foster the involvement of all the 42 participants and the duration was not understood as a limitation for expressing their perceptions freely. All the focus groups but one were recorded, with the authorization of all the participants.

The focus group participants were volunteers and were recruited from among both BC and WC employees. In addition, we made a point of recruiting members from some of the governance and other elected social bodies (due to their knowledge of the governance channels). In total, 24 BC employees and 16 WC employees participated, 8 of the total belonged to governance bodies. Two participants chose not to give information about their professional status or whether or not they were members of any of the cooperative’s bodies.

To encourage debate and discussion among the participants, they were given a brief questionnaire prior to the focus group (see Annex 1). The questionnaire is a quantitative method of data collection in which the perceptions of the participants about the subject under analysis are recorded in a quantitative manner. The questions in the questionnaire designed at MIK, the management research center in Mondragon University, were approved by the company’s HRM department.

The questionnaire consisted of eight items and one control question. The items were related to perceptions about PDM at the three levels identified in the literature (in the individual’s job, in the department/unit/section, and in strategic decisions). The items also enquired about perceptions of the psychological consequences of COVID-19 in their lives. In the survey, a variety of answers was provided in order the participants to understand the questions. Lastly, there was a control question related to the professional circumstances of the employee; namely, whether he/she was BC or WC, and whether or not they formed part of one of the governance bodies.

The questionnaire was completed by all the employees who participated in the focus groups and thus served as a starting point for the analysis of their perceptions. The responses of the questionnaire were visualized on a screen and analyzed by both participants and researchers, thus stimulating a dialogue with which to probe, question by question, the quantitative responses and understand the employee’s perceptions in a qualitative way. This dynamic led participants to express perceptions and feelings beyond the provided questions and answers.

In respect of the analysis, a descriptive analysis of the quantitative data was done. In relation to the qualitative data, the recorded focus groups were transcribed and the notes taken in the non-recorded focus group were included in the transcription. Then, the data was organized by each of the research questions. And finally an interpretation of the results was done in order to understand the impact of the COVID-19 in UAS.

In short, we decided to use a methodology that was both quantitative and qualitative in this research. Quantitative research allows us to determine the relation between elements, but it does not provide information about the *hows* and *whys*, which permit a qualitative point of view (Villarreal Larrinaga and Landeta Rodríguez, 2010), and which is the aim of this article.

## Results

### Research Question 1

Research question 1 asked how COVID-19 has affected employees’ perceptions on PDM and how governance and communication channels have been adapted to satisfy the new needs arising out of the current situation. Table 1 summarizes the quantitative results for Research Question 1 for the overall sample as well as for the both groups (WC and BC separately). The results related to PDM have been classified according to the three levels cited above: in the individual’s job; in the department/business unit; and in strategic decisions. We have also included in the Table 1 the results regarding the perceptions about IC. As said before, both concepts are linked in our reality.

**TABLE 1:** Perception about PDM and IC. Percentage per each job-professional group.

	To what extent has COVID-19 affected your perception of…											
	PDM in your job?			PDM in your department/business unit?			PDM in strategic decisions?			IC?		
	WC	BC	Total	WC	BC	Total	WC	BC	Total	WC	BC	Total
Considerably	7.0%	4.3%	5.13%	7.0%	9.5%	8.11%	23.0%	8.7%	13.16%	37.5%	58.3%	50.0%
Somewhat	40.0%	13.0%	25.64%	27.0%	0	10.81%	8.0%	26.1%	18.42%	43.8%	29.2%	35.0%
Slightly	20.0%	56.5%	41.03%	27.0%	42.9%	35.14%	15.0%	21.7%	18.42%	6.3%	8.3%	7.5%
No effect	33.0%	26.1%	28.21%	40.0%	47.6%	45.95%	54.0%	43.5%	50.00%	12.5%	4.2%	7.5%

When participants answer the questions about IC, they refer to both governance channels and day-to-day communication channels. The participators themselves link IC with PDM.

It is interesting to observe that, while only 28.21% of the employees think that COVID-19 has not affected their level of PDM in their job, this percentage rises to 50% in the case of strategic decisions. However, it is also of note that, while only 5% think that the crisis has affected their PDM in job considerably, this proportion doubles in the case of strategic decisions. In this way, there seems to be different perceptions about PDM according to the level.

Notwithstanding the above-mentioned, we must highlight that, in general, when asking the employees about their perception related to IC, they perceive that it has been affected somewhat or considerably by COVID-19 (more than 80% in both cases).

If we focus on PDM in the job, the predominant perception among BC employees is that COVID-19 has had only a slight effect (56.5%), while less than half that percentage of WC employees have the same opinion (20%). On the other hand, among the WC employees, 47% conclude that COVID has had a considerable (7%) or intermediate (40%) influence, in contrast to 17.3% of the BC employees. Therefore, it can be observed that the impact of COVID-19 has more affected the WC employees in their perceptions of PDM in the first level.

In terms of PDM in the department/business unit, the percentages of employees that perceive considerable changes are low. It is noticeable that 27% of WC employees identify an intermediate effect (somewhat), while a large percentage of BC employees consider that the effect has been slight or null. In addition, it is noteworthy that the perception among BC employees that COVID-19 has had a slight effect is around 15 points higher than that of their WC colleagues.

In relation to strategic decisions, the prevailing perception among both BC and WC employees is that COVID-19 has not affected PDM. More than half the WC employees and almost half the BC employees perceive no effect. However, it is noteworthy that, among the former group, 23% consider COVID-19 to have affected PDM in strategic decisions considerably, while 26% of the latter group perceive the effect to be intermediate, both of which percentages exceed those attributed to other levels. This implies a disparity of perceptions among employees, even among those belonging to the same category (in this case, WC).

When we explore PDM in their job further, the employees related this sensation with a reduction in interpersonal communication and the impact of COVID-19 on their day-to-day job. In this sense, in the feelings expressed by the workers, it seems that something has been lost in this communication, and therefore, in their PDM.

*“participation*…, *to a certain extent it has affected us*… *Not in a serious way, but yes*…*I’ve been here practically every time, but there was always somebody missing*…*either they aren’t here*…*they don’t log on, or they can’t log on*… *that kind of thing*… *if you aren’t near*…”

*“some have been (working) at home, and although the telephone is there, it just isn’t the same”*

*“I haven’t been on site: I might have been there to make decisions*… *meetings via Skype*…*a lot of e-mails”*

Therefore, we can derive that when employees perceive that job participation has been affected considerably (5.13%) or quite a lot (25.64%), this change is perceived negatively.

In the department/business unit, on the other hand, PDM was related to modifications in the way of working. In this case, there is a high percentage of employees that perceives that PDM was slightly affected (35.14%) or not affected (45.95%). This result reflects that the COVID-19 has not affected the perception of employees in terms of PDM itself, but it has affected the type of decisions that had to be taken in order to face the economic situation derived from the COVID-19.

*“for example, in my area*… *Decisions have had to be taken up until now that had practically never been taken*… *there were some things that we didn’t do as a rule”*

*“a lot of projects have been paralysed, visits to quarries to check materials, etc. all these processes have been paralysed. In the department we have had to redistribute positions*…”

In UAS, as in other firms, diverse types of meetings serve as channels of communication. In this case, when asking about IC the participants mentioned meetings of collaborators and departmental meetings, both of them related to employee PDM in department/business unit.

In this context, BC employees perceive the need to maintain or to resume monthly meetings that were called off because of COVID-19. The absence of these meetings means they have not had access to information.

*“the fact is we don’t receive information”*

*“there is some information, but in comparison to before there is a real lack of information”*

*“if there is information, it doesn’t reach us”*

*“the information is incomplete; we receive a document with the most important points, which are usually about results. At the social level, before there was much more extensive information than what you can send now in a document.”*

Some of the BC employees feel there should be more information, with the same structure and of the same type as that which existed before COVID-19: “*Yeah, yeah, they need to hold the meetings, the same as before.”*

In the case of WC employees, departmental meetings have not taken place in all departments/sections, but in some cases they do not know if this is due to COVID-19 or other reasons.

*“in the department we belong to, the last two meetings haven’t taken place, but I’m not sure if it’s because of this or not”*

*“we’ve had meetings, different to before, but we have had them. It isn’t the same, but, well*…”

“…*yes, we should have the monthly meeting, me and my department, and we don’t do it.you can see there that*…*somebody will say, man, we need to talk*…”

When we enquired about the perception of their voice having been heard by their direct boss during COVID-19, differences were apparent between BC and WC employees. The majority of participants felt they had been heard by their direct boss during the pandemic, but the extent varied, with more positive perceptions among WC employees:

*“I’ve felt listened to”*

*“in my case, with my boss, as he’s an individual person, it’s easy, but when it’s a team I think it will be more and more difficult”*

*“with COVID I think even more, I reckon*…”

*“there has been more communication with the direct boss”*

*“I always do, because I have a close relationship*…*We work alongside each other every day.”*

However, negative perceptions were also expressed: *“well, not in every case*…*personally, I do, but I know there are some people who feel somewhat abandoned, people who were (working) at home”.*

In the case of BC employees, their perceptions were generally negative:

*“I miss the monthly meetings, where we talk about the line*…*it’s a forum for discussion*…*yeah, when I’ve had something to say I’ve gone, and yes, I’ve been listened to, but it isn’t a natural forum*…*as a team, as a group*…*Things that go on in the line are spoken about in the bi-monthly meetings, and seeing as they aren’t being held*…*a lot of things aren’t discussed.”*

*“we can’t feel listened to all the time on the production line, because there’s a single person responsible for 20 people, so it’s understandable that you don’t feel listened to at certain moments because one person can only do so much*…*but this hasn’t got worse during the COVID-19 period”*

*“In production, it’s because of lack of time*…. *Because we can’t leave our work post, neither you nor he”*

Thus, in this case, we can conclude pointing out that the perception about PDM in department has been neither positive nor negative. The perceptions are neutral. However, regarding to the IC related to the department/business unit, most of the evidence comes from the BC employees, practically all of which is negative, with regard to the holding of meetings, the absence of channels, the lack of information and the perception of being heard. There is no evidence from WC employees other than a positive feeling regarding listening from their leaders.

Lastly, when we asked employees about their perception of their PDM in strategic decisions, they also referred to changes derived from COVID-19, as in the case of the PDM in the department/business unit:

“… *and then also the decisions, you know*,…*there are decisions that have been put on hold because of COVID-19”*

*“the whole aspect of investments*… *what was planned has been postponed”*

*“even the management plan, it was changed after it having being agreed on 1*^st^* January”*

In the focus groups there was talk of the Governing Council, and the fact that it had to adopt decisions of major importance that would not have been taken pre-COVID-19.

*“in the governing council, we have made decisions*… *it has been*…*.”*

*“the situation with COVID has led us to engage in a series of important decisions, talks, assemblies*… *it has meant extra work, much more than during normal times*…”

In the more institutional sphere, there is a general negative perception related to the lack of celebration to the social council constituents’ meetings among the BC workers. Indeed, they require to hold these meetings, because of the interaction they facilitate. They even propose ideas to celebrate them, in smaller groups. In the case of WC, they propose to hold these types of meetings following a different format, maintaining the pre-COVID-19 structure.

*“face-to-face in the end is the same as on paper, but if you have any queries, you have someone to ask, they clear up any doubts you might have there and then*…”

*“I sincerely believe that, the way things are now, several of the meetings, with the people who usually attend and everything, they could easily be held here*…*not the very big groups, but the rest could be”*

*“not only insofar as the information you receive, but also when it is given*…”

*“for me it’s different if they explain things to you or*… *there isn’t any interaction, questions and the rest”*

*“I’ve heard that people feel much more comfortable if the meeting is in person”*

Additionally, some participants proposed mechanisms that could be used to allow the information to be transmitted satisfactorily. In the BC workforce, they proposed physical meetings per line or shift “*perhaps there needs to be a meeting for every shift/line*”. In the WC workforce, on the other hand, they suggested using the on-line format because the employees have the necessary resources “*I think that the social council constituents’ meetings can be held by Skype, for example in the offices*,” “*yeah, they are missed*…*people feel much more comfortable holding social council constituents’meetings.*”

In terms of how PDM in said meetings had changed, part of the WC employees indicated that attendance was low, though the reason was unknown (among the BC employees, it is compulsory to attend these meetings because the production line stops because of it).

With respect to informative talks (pre-General Assembly), which were held in smaller groups, one of the members of the Social Council affirmed that the Council had come to the conclusion that PDM had improved.

This affirmation is reflected in the generally positive evaluation by employees of the General Assembly; the fact it had taken place on-line, and that there had been informative talks given prior to the event, in which almost the entire content of the Assembly could be downloaded, was considered to have facilitated understanding.

*“it was more positive*,…*especially the key points that were to be voted on, much better than arriving and*…”

*“I think that it has been rated very well in general”*

*“personally, I think it went very well”*

*“there were debates, and those debates don’t always arise in an assembly”*

It seems, therefore, that workers perceive differences between the governance channels above explained. Thus, for the most operational and frequently used channels, such as social council constituents’ meetings, it seems that BC employees perceive that they miss holding these meetings and they require to hold them, while WC ask for changes in the way they are held. So the general impression is that PDM in this channel can be improved and/or modified.

On the other hand, the most important channel of strategic participation, the General Assembly, is valued positively, and this positive perception is related to the effort made by the cooperative to carry it out online, after holding informative talks in small groups.

In conclusion, we found evidence that COVID-19 has influenced the perceptions of PDM at the three levels explored: in the individual’s job, in the department/business unit, and in strategic decisions. Such perceptions are positive or negative depending, on the one hand, on the job-professional group (BC-WC) and, on the other hand, on the level of participation and the governance and communication channels.

### Research Question 2

Secondly, we asked the employees about how COVID-19 had affected some psychological outcomes, in particular, their satisfaction, motivation, commitment or trust, requesting that they identify the outcome which had been most negatively and positively affected. The results are shown in Table 2.

**TABLE 2:** Impact of COVID-19 on psychological outcomes.

	Which of the following outcomes has been most NEGATIVELY affected for you during COVID-19?	Which of the following outcomes has been most POSITIVELY affected for you during COVID-19?
Satisfaction	26.0%	5.0%
Motivation	21.0%	14.0%
Commitment	0.0%	45.0%
Trust	19.0%	14.0%
No answer	33.0%	21.0%

Regarding the outcomes that employees perceive to have been most negatively affected, more than a third of those questioned did not answer, as they considered that none of the possible responses represented the outcome they personally felt had been the most NEGATIVELY affected during the period of COVID-19, or because, in their opinion, no outcome had been affected negatively.

*“I haven’t voted because I haven’t recognized any of those*…*I don’t know, personal uncertainty*…”

*“I don’t think any of them have”*

*“in my case, it hasn’t affected me negatively”*

*“here, internally, I don’t think it has affected us as much as we thought it was going to affect us*…”

Satisfaction, motivation and trust (in that order) have been the outcomes most negatively affected, with similar percentages among the three. It is noteworthy that nobody identified commitment.

*“I’ve chosen satisfaction because of how uncomfortable it is to wear a facemask”*

*“I chose satisfaction too*…*apart from the sense of insecurity that COVID creates in normal life, to tell the suppliers that you aren’t going to be able to do it because you’re afraid and to say no to them, delay things, well, that isn’t satisfactory.”*

*“I said satisfaction. because the satisfactions you might have had before, they’ve been taken away*… *well, they haven’t been taken away, but they’ve been diluted a bit or put to one side”*

*“in the beginning it was scary, the threat of being let go*…*but in the end it didn’t turn out to be the case”*

*“I chose motivation, you got up every day without knowing exactly what to expect, what you were going to find. the situation has been complicated and to be motivated*…*is complicated”*

*“I voted for trust, because the situation creates distrust and insecurity, not only at work*…*24 hours a day”*

*“I voted for trust, in part because of the uncertainty of not knowing if the guy you have next to you is going to be ok, if you’re going to be all right, that fear, a little*…”

*“I chose security, because of the present situation, because of the health threat*…*economic too, but more because of health”*

In the qualitative part of the focus groups, some of the participants who did not answer mentioned uncertainty as the outcome that had been most negatively affected for them.

*“there’s a lot of uncertainty there*…*to do with everything, economic, personal*…”

*“initially we did feel a lot of uncertainty about how and when it was going to bring pressure - at the business level (meaning that expectations as a company at the beginning were bad, but time has shown that expectations weren’t so bad)*

*“I also voted for uncertainty in a generic way, maybe tomorrow we’ll all be sent home and have to wait to see what happens*…”

With respect to the positive perception of the pandemic, 45% of the employees considered that their level of commitment had been the clearest outcome.

*“In this sense, I’ve also chosen commitment, because I think it has been demonstrated by actions*…*they cancelled the contract of several temporary employees and they made a commitment to contract them again and they did; the company committed itself to a series of people that were going to be temporary members later on, and they did that too*…*so, on the part of the organization, a series of commitments were undertaken and fulfilled”*

*“from the outset, we gave ourselves 85%^3^. Once more, our commitment has been firm*… *and in general we have committed ourselves fully to complying with the safety measures that have been implemented”*

*“in terms of remote working, I think the commitment of the employees and the mutual trust among employees and as a team have been enormous*…”

*“really in a situation where you are confronted with these difficulties, the urge to lend a hand*…”

*“yeah, the commitment to help one another*… *it’s at difficult times like these that you can see it*…”

*“in the end we were in a situation that was so*…*one day we had to come in, the next day we had to go to the post office, the next day*…*you did things that*…*I don’t know how to explain it, you just had to muck in”*

*“I voted for commitment*…*it was like that before, you know, but now there’s more commitment, for safety’s sake, because of what could happen*…”

*“I picked commitment too because as individual people and a cooperative we are committed to eradicating this pandemic, everyone in his/her own way, and also as a cooperative, using our resources, showing solidarity with our colleagues*…”

Motivation and trust were also outcomes the participants felt had been most positively affected in their case.

*“trust, in the sense that the work will get done even though the employees aren’t there physically”*

*“I had chosen trust, but they (motivation and trust) were also factors*…*maybe more in the company, and it was a general comment*…*It’s when there are blows like this that you realize the value of the company you work for*…”

*“insofar as remote working, I think we’ve been able to see that the commitment of the employees and the mutual trust amongst them and as a team has been considerable*…”

*“I’ve felt motivation to come to work every day, whenever we could come, the fact that we still have a job*…*that’s good motivation”*

However, in this case too, 22% of those surveyed did not respond, as they considered that none of the possible answers aligned with the factor they personally felt had been most POSITIVELY affected during the COVID-19 period. Empathy was mentioned as another outcome that had varied positively.

*“and something that was not among the answers and that for me has been very enriching is that, in our team, there are people who work remotely, and we have put ourselves in their shoes and we’ve learned about the implications of other forms of work, and it has been a way of empathizing, totally”*

Another topic that was addressed among the participants was the emotions experienced during the COVID-19 period. In this regard, we observed a disparity in the results. For example, 14 people experienced fear as employee-members during COVID-19, while 13 lived through it in a calm way.

There was talk of tranquillity in both the economic-business and personal spheres.

*“from almost the beginning we could see that, as soon as things got going again there was demand from the market, so in the end that was calming; and, in terms of the work itself, it was evident that the work was getting done*… *from a lack of tranquility, which there might have been in the days when we didn’t really know what was happening*…*it lasted a very short time*…*there was tranquility because all the data proved things were going well”*

*“I’ve been calm enough. at home the norms have been complied with, and at work too*…”

In contrast, the employees who recognised having experienced fear were referring to the personal, family sphere.

*“I was scared because this is such a shocking thing that has come out of nowhere that*…”

*“I was afraid and stressed, because living in such a small apartment, with the kids, not being able to go and see the grandparents*,… *it was crazy”*

*“fear of the unknown*… *in the end everything was scary”*

*“I was afraid, but because fear is irrational*… *and then there was the uncertainty of each day*… *to cope with uncertainty is very complicated”*

*“yes, I also felt afraid, and anxious*…*to a certain point yes*…*most of the day I was calm, but yes*…”

*“with me it’s fear, it’s not that I feel mentally blocked, but*…*perhaps it’s more apprehension, but*…”

Those that chose anxiety or stress were generally referring to their personal life, rather than work.

*“I’ve chosen anxious because of the whole situation we had with construction projects*…*plus, you had to go out, you were on site with 100 people who had travelled there on public transport*…*and then you went home and*…*you’d think to yourself, “What do I do? Do I change out of my clothes before I go in?”*…*I know, you’re not in a hospital, but in the end*…*you ask yourself “Could I have been infected?”*

*“in terms of work*… *stressed”*

*“I’ve been anxious*…*in general, a bit, with the family*,… *but I also have relatives who live alone*… *and that creates anxiety. Or it could be fear too*…”

Again, some of the people who had not voted said they had experienced the COVID-19 period with uncertainty and with a sense of not knowing what to expect.

*“I’d say it was uncertainty with me*…”

*“I put fear, but you don’t really live in fear, it’s uncertainty, really. More than anything because of those around me, not me personally*…*I’ve had that worry, it isn’t fear. If I chose fear, it’s more to do with personal factors.”*

*“I didn’t vote, for example, because I haven’t been anxious, or afraid*…*I’ve been a bit on edge about what was happening”*

In conclusion, it seems that satisfaction, motivation and trust are outcomes that have been valuated more negatively during the pandemic. Some of the participants mentioned the uncertainty and the anxiety suffered when talking about psychological outcomes. In relation to the most positively valuated psychological outcome, the one that stands out is the commitment: commitment among colleagues, commitment with the economic situation of the cooperative, solidarity with colleagues and with the cooperative itself.

With regard to the emotions that arose during the period, some people experienced it with tranquility, while others said that they had experienced fear, but mainly in relation to the family environment and not to the work environment.

## Discussion

At both the qualitative and quantitative level, we have found evidence that COVID-19 has affected differently employees’ perceptions of their PDM on three levels: in their individual job, in their department or business unit, and in strategic decisions. The distinction among the levels of the PDM settled by Bernstein (1976b), Albizu et al. (2005), Baniandrés and Larrinaga (2013) and Eurofound (2013) is appropriate as it has been observed that COVID-19 has affected such levels in a differentiated manner. In this regard, although the majority of employees perceive that COVID-19 has had a slightly or no effect in their PDM, it is striking that the 80% of the employees perceive that IC, that includes channels where employees participate, has been considerably/somewhat affected. Therefore, we could conclude stating that COVID-19 has affected PDM fundamentally through the communication channels.

In UAS, participation in one’s job has been related with interpersonal communication. And the perceptions gathered in the focus groups can be interpreted as negative, where there is a lack of relationship, and therefore interpersonal communication. In this sense, in the job level, the participants linked their PDM with the digitalization and remote working imposed by COVID-19 (DeFilippis et al., 2020).

Regarding to the department/business unit and in particular, BC employees, taking into account that the monthly meetings are not held, employees perceive that the absence of channels leads to a perception of a lack of information. Moreover, there is a feeling of not being heard due to the lack of effective communication channels with those responsible. We can conclude that due to these circumstances, the perception of PDM seems to have been deteriorated.

Referring to strategic decisions, among others, Wegge et al. (2010) and Weber et al. (2020) suggest that employees can participate directly or indirectly, through representative channels, in the organisations. In this regard, the participants perceive that there is a need to improve the closest and most operational and frequently used channel, such as the social council constituents’ meeting, especially from the point of view of the BC workers. Employees miss such meetings. Indeed, among other channels, information related to the cooperative is transmitted via the social council constituents’meeting, and if such meetings are not held, the possibility of interaction and participation is lost.

However, the holding of informative talks in smaller groups has led to the conclusion (by the Social Council) that participation in certain channels has improved. There is a positive perception of the effort made by the cooperative to hold the General Assembly online after holding small informative talks which has strengthened participation in the cooperative’s highest decision-making body. The strengthening of participation of members of the cooperative in the supreme decision making body, where each member has one vote regardless the contribution made, supports the idea that the main democratic channel has to function even in a pandemic and uncertainty context. Thus, in UAS, worker-members could participate in organizational and governance processes (Harrison and Freeman, 2004) also during the COVID-19 era.

Moreover, it can be said that the perception of PDM is directly connected with the celebration, or not, of both governance channel meetings and day-to-day meetings (e.g., social council constituents’meetings, informative talks, department/business unit meetings…); in other words, with the efficiency of all these channels.

In conclusion, in this case, we have found evidence that the PDM in certain channels has deteriorated due to COVID-19 and this conclusion can be linked with the degeneration thesis that is being discussed within the cooperatives. However, in this particular and exceptional situation, UAS has implemented regeneration initiatives in order to celebrate successfully the meeting of the supreme body of the cooperative, that is, the General Assembly. Moreover, the employee-members also make some proposals to foster PDM in this COVID era that can be seen as regeneration initiatives. Therefore, as it stated in Bretos et al. (2020), degenerative and regenerative tendencies can occur simultaneously in the cooperative because as demonstrated, the cooperative has mechanisms and resources to revitalize democracy.

Furthermore, on the three levels, perceptions about the effect of COVID-19 on PDM are manifested as a disparity of opinions, and belonging to one labour category or another – BC versus WC – is a defining element. Therefore, job classification is a key point as in Hu et al. (2010), Arregi et al. (2018), and Belategi et al. (2019).

When employees were questioned about the psychological outcomes most negatively affected by COVID-19, there was not a predominating outcome. However, it is worth mentioning uncertainty as an outcome highlighted by those who did not identify with any of the proposals (satisfaction, motivation, trust). In this sense, we should underline that this outcome is not a trivial one in the era of COVID-19, as studies (Rettie and Daniels, 2021; Reizer et al., 2021) indicate that intolerance to uncertainty has constituted a significant risk factor for mental and psychological distress during the COVID-19 outbreak.

With respect to the outcomes that have been affected positively, there has been a clear boost of commitment among the employees, which is linked to the effort that each person has made within the company as a result of the pandemic. The commitment of employees in the current situation derived from the COVID-19 outbreak has become one of the utmost prominent primacies for human resource departments (Chanana, 2020); in fact, commitment has been shown to foster employee performance (Adhitama and Riyanto, 2020). Although commitment is not one of the first outcomes to take into account when preparing for and combating against the new challenges raised by the pandemic, it is relevant to highlight that the academic sphere has demonstrated a correlation between the commitment of employees and mental health, causing for example insecurity, confusion, emotional isolation and stigma for employees in times of uncertainty (Pfefferbaum and North, 2020). In the present case study, in contrast to that shown by other research (Jung et al., 2021), there is an evident perception among employees that COVID-19 has not had negative effects on commitment, and that, rather than deteriorating, it has been strengthened. The commitment among colleagues, with the economic situation of the cooperative, solidarity with colleagues and with the cooperative itself shown by participants can be a sign of robustness and regeneration, since socially-oriented targets prevail over profit ones. One of the possible reasons that can explain the positive perception of commitment is that UAS has guaranteed, as far as possible, workplace democracy.

Lastly, with respect to their experience as employees/employee-members during COVID-19, those who mentioned “fear” have done so from a family/personal perspective, while those who have identified “anxiety” and “stress” as outcomes have generally done so with respect to work. In contrast, a perception of tranquillity has been transmitted from both the professional and personal spheres. According to the literature available, all these feelings and emotions are fruit of the pandemic. Indeed, as a result of the changes brought about by COVID-19, employees’ anxiety, stress (Rossi et al., 2020; Sahni, 2020), risk perception (Baldassarre et al., 2020; Dryhurst et al., 2020; Wise et al., 2020), and anxiety regarding isolation, stigma and discrimination have all increased significantly. Evidence shows that this stress has been caused by new factors, such as the threat represented by the virus to health and life itself, the restrictions and recommendations implemented by the authorities (home confinement), isolation and lack of social support (Irigoyen-Camacho et al., 2020).

## Conclusion

In this article, with the aim of filling in the gaps detected in the theoretical framework, we have employed the case study methodology and analyzed the quantitative and qualitative data that it provides. Although we do not claim to be able to generalize regarding the results and conclusions, as each company/business has its peculiarities and nuances, the data obtained as a result of our research are relevant in terms of constructing arguments, forming interpretations and gaining insight into experiences, and can be of use to future research in democratic enterprises in this new era.

In our case study, PDM has been affected by COVID-19. With regard to the day-to-day meetings and governance channels, although there are improvements and changes that still need to be implemented, the cooperative has responded well by continuing the work of its most important body, the General Assembly, with positive results.

The findings reveal differences between BC and WC employees that are proof of the need for academics to perform research that seeks to explain employees’ perceptions with respect to the work they do and how they participate.

It is well-known that academics have studied the phenomenon of MONDRAGON and that exists a discussion about the dilemmas and paradoxes arising from the PDM (e.g., Greenwood and González, 1991; Stohl and Cheney, 2001; Cheney, 2006; Azkarraga et al., 2012; Agirre et al., 2014; Errasti et al., 2016, 2017; Bretos et al., 2017, 2020). With this article, we provide another evidence to this discussion about the PDM in MONDRAGON. Our research has found, on the one hand, that degeneration and regeneration tendencies can occur simultaneously in a cooperative and on the other hand that UAS has mechanisms to regenerate the workplace democracy. The study has also focused, in the different perceptions of WC and BC, topic that had not been included in the previous literature.

Our psychological results revolve around commitment to the cooperative and uncertainty. The positive effect of this situation on perceptions of commitment is of note. An interesting line of research in the future would be to analyze if this is a specific effect of this company, of cooperatives in general, or of companies that have resumed their activity following an initial period of closure. And in case this phenomenon applies to other companies, it would be interesting to research about which are the antecedents and consequences of it. With respect to uncertainty, this outcome is more associated with life in general, and with the global climate of uncertainty, than with the work sphere specifically.

The health crisis has represented a turning point in the field of human resources and has made patent once more the need to treat employees as a central and fundamental element of an organization. COVID-19 has implied a change in our understanding of employee participation, an important and central aspect in democratic organizations.

## Data Availability Statement

The raw data supporting the conclusions of this article will be made available by the authors, without undue reservation.

## Author Contributions

All authors listed have made a substantial, direct, and intellectual contribution to the work, and approved it for publication.

## Conflict of Interest

The authors declare that the research was conducted in the absence of any commercial or financial relationships that could be construed as a potential conflict of interest.

## Publisher’s Note

All claims expressed in this article are solely those of the authors and do not necessarily represent those of their affiliated organizations, or those of the publisher, the editors and the reviewers. Any product that may be evaluated in this article, or claim that may be made by its manufacturer, is not guaranteed or endorsed by the publisher.

## Footnotes

1 The social functions of the Governing Council are related with employee-members (new contracts, canceling of contracts, leave of absence, professional qualifications, rewards…). It interprets the cooperative’s statutes and Rules of Procedure in the case of doubts and proposals by the Assembly to modify the statutes or procedures, and it represents the cooperative before third parties. It carries out a monthly control of the cooperative’s economic activity, presents the annual report and end-of-year balance sheets and proposes the allocation of surplus to the General Assembly, among other financial functions (Altuna and Grellier, 2008).

2 A work schedule that varies depending on demand and which adapts to the needs of the company.

3 In this case the participant refers to a payroll advance (a payment option within cooperatives). The percentage of salary to be received can be lowered if agreed by the General Assembly as a measure to counteract or improve the economic situation of the cooperative.
