# Supplementary material for: Employee Perceptions About Participation in Decision-Making in the COVID Era and Its Impact on the Psychological Outcomes: A Case Study of a Cooperative in MONDRAGON (Basque Country, Spain)
Source: Front Psychol. 2022 Feb 4;13:744918. doi: 10.3389/fpsyg.2022.744918 (PMC8854208; doi:10.3389/fpsyg.2022.744918)
Supplement: Supplementary file 1 [file Data_Sheet_1.PDF]

Supplementary Material

1 Questionnaire

1. Are you a blue collar worker or a white collar worker? Are you a member of any governance body?

a) I am a blue collar worker and I do not belong to any governance body
b) I am a blue collar worker and I belong to a governance body
c) I am a white collar worker and I do not belong to any governance body
d) I am a white collar worker and I belong to a governance body

2. To what extent has COVID-19 affected your perception of internal communication in ULMA ARCHITECTURAL SOLUTIONS?

a) It has not had effect
b) Slightly
c) Somewhat
d) Considerably

3. Have you felt that your voice has been heard by your responsable during COVID-19 era?

a) No, I have not felt heard
b) I have felt heard on some occasions
c) I have felt heard always

4. To what extent has COVID-10 affected your perception of participation in decision making in your job?

a) It has not had effect
b) Slightly
c) Somewhat
d) Considerably

5. To what extent has COVID-10 affected your perception of participation in decision making in your department/business unit?

a) It has not had effect
b) Slightly
c) Somewhat

d) Considerably

6. To what extent has COVID-10 affected your perception of participation in decision making in strategic decisions?

a) It has not had effect
b) Slightly
c) Somewhat
d) Considerably

7. Which of the following outcomes has been most NEGATIVELY affected you during COVID-19?

a) Satisfaction
b) Motivation
c) Commitment
d) Trust

8. Which of the following outcomes has been most POSITIVELY affected you during COVID-19?

a) Satisfaction
b) Motivation
c) Commitment
d) Trust

9. Emotionally, how did you experience the COVID-19 era?

a) Anxious
b) Afraid
c) Calm
d) Stressed
